# Causal Effects of Genetically Predicted Cardiovascular Risk Factors on Chronic Kidney Disease: A Two-Sample Mendelian Randomization Study

**DOI:** 10.3389/fgene.2019.00415

**Published:** 2019-05-03

**Authors:** Hui-Min Liu, Qin Hu, Qiang Zhang, Guan-Yue Su, Hong-Mei Xiao, Bo-Yang Li, Wen-Di Shen, Xiang Qiu, Wan-Qiang Lv, Hong-Wen Deng

**Affiliations:** ^1^Center of System Biology and Data Information, School of Basic Medical Science, Central South University, Changsha, China; ^2^Center of Reproductive Health, School of Basic Medical Science, Central South University, Changsha, China; ^3^Kangda College of Nanjing Medical University, Nanjing, China; ^4^College of Public Health, Zhengzhou University, Zhengzhou, China; ^5^Institute of Biomedical Engineering, West China School of Basic Medical Sciences and Forensic Medicine, Sichuan University, Chengdu, China; ^6^Tulane Center of Bioinformatics and Genomics, Department of Biostatistics and Data Science, Tulane University School of Public Health and Tropical Medicine, New Orleans, LA, United States

**Keywords:** two-sample mendelian randomization, genome-wide association study, cardiovascular risk factors, chronic kidney disease, causation

## Abstract

Observational studies have demonstrated that cardiovascular risk factors are associated with chronic kidney disease (CKD). However, these observational associations are potentially influenced by the residual confounding, including some unmeasured lifestyle factors and interaction risk factors. Two-sample mendelian randomization analysis was conducted in this study to evaluate whether genetically predicted cardiovascular risk factors have a causal effect on the risk of CKD. We selected genetic variants associated with cardiovascular risk factors and extracted the corresponding effect sizes from the largest GWAS summary-level dataset of CKD. Cardiovascular risk factors contain high density lipoprotein (HDL) cholesterol, low density lipoprotein (LDL) cholesterol, total cholesterol (TC), triglyceride (TG), glycated hemoglobin (HbA1c), fasting glucose, systolic blood pressure (SBP) and diastolic blood pressure (DBP). A Bonferroni corrected threshold of *P* = 0.006 was considered as significant, and 0.006 < *P* < 0.05 was considered suggestive of evidence for a potential association. Genetically predicted DBP was significantly associated with CKD [odds ratio (OR) was 1.35 (95% confidence interval (CI) (1.10, 1.65); *P* = 0.004)]. There was suggestive evidence for potential associations between genetically predicted higher HDL cholesterol [OR: 0.88, 95%CI (0.80, 0.98), *P* = 0.025] and lower adds of CKD, and between higher SBP [OR: 1.36, 95%CI (1.07, 1.73), *P* = 0.013] and higher adds of CKD. However, genetically predicted LDL cholesterol, TC, TG, HbA1c, and fasting glucose did not show any causal association with CKD.

## Introduction

Chronic kidney disease is a global-health challenge affecting at least 225.7 million people in the world, especially in developing countries, in 2010 (Hill et al., 2016). The prevalence rates of CKD have dramatically increased in the past several decades, which threaten human health seriously (Romagnani et al., 2017). Observational studies have detected a close relationship between increased risk of cardiovascular diseases and CKD, showing that major cardiovascular diseases account for approximately 50% of the causes of death in CKD patients (Heywood et al., 2007). Multiple cardiovascular risk factors, including lipids (Hager et al., 2017), glycemic traits (Ceriello et al., 2017), and blood pressure (Hall et al., 2014; Ceriello et al., 2017), have been reported associated with CKD in observational studies. However, a review reported a reverse effect of CKD on hypertension and dyslipidemia (Schiffrin et al., 2007), demonstrating that CKD can promote hypertension and dyslipidemia, which in turn can lead to the progression of renal failure (Schiffrin et al., 2007). The reverse association between cardiovascular risk factors and CKD might be influenced by potential confounders of unmeasured lifestyle factors.

Mendelian randomization analysis, analogous to randomized controlled trials (RCTs), has been widely performed to investigate the potential causality between genetically predicted environmental factors and diseases (Katan, 2004; Lawlor et al., 2008; Boef et al., 2015). As the genotype is randomly assigned during the meiotic process, MR analysis results will not be distorted by confounders (Emdin et al., 2017), a major limitation of traditional observational studies. Compared with one-sample MR, which extracts the effect estimates for IV-exposure association and IV-outcome association from the same sample, TSMR estimates that these associations in different samples and the estimates are then combined to infer the potential exposure-outcome causal association (Davey Smith and Hemani, 2014).

Recently, a TSMR analysis demonstrated that higher HDL cholesterol concentration was causally associated with better kidney function, while LDL cholesterol or triglyceride concentration showed no association with kidney function (Lanktree et al., 2018). Another MR analysis detected that genetically determined type 2 diabetes (T2D) was causally associated with decreased eGFR in populations of Chinese aged above 40 (Xu et al., 2016). However, no studies have been conducted utilizing TSMR to summarize the causal relationship between cardiovascular risk factors and CKD. Therefore, in this study, we performed TSMR analysis to examine the causal effect of cardiovascular risk factors, including lipids, glycemic traits, and blood pressure on CKD, based on GWAS summary-level data.

## Materials and Methods

### Data Sources

We selected genetic variants associated with cardiovascular risk factors, including lipids, glycemic traits and blood pressure, and then extracted the corresponding effect sizes for CKD using the largest GWAS summary-level dataset (Dupuis et al., 2010; Soranzo et al., 2010; Willer et al., 2013; Ehret et al., 2016; Pattaro et al., 2016). No ethical approval was conducted in our study due to this being a re-analysis based on previous collected and published data. Lipids data of HDL cholesterol (*n* = 187, 167), LDL cholesterol (*n* = 173, 082), TC (*n* = 187, 365), and triglyceride (TG, *n* = 177, 861) were extracted from the GLGC consortium (Willer et al., 2013). We obtained glycemic traits data of fasting glucose (Dupuis et al., 2010) (*n* = 133, 010) and HbA1c (*n* = 46, 368) (Soranzo et al., 2010) from the MAGIC consortium. And blood pressure data (Ehret et al., 2016) of SBP (*n* = 317, 754) and DBP (*n* = 317, 756) were extracted from UK Biobank imputed genotype data^1^. Additionally, people with eGFR based on serum creatinine (eGFRcrea) < 60 mL/min/1.73 m^2^ were defined as CKD, and the dataset were acquired from CKDGen consortium (*n* = 117, 165) (Pattaro et al., 2016). All of the dataset were conducted on European populations from RCTs and population-based cohorts. Genomic control to each sample was applied to correct for inflated test statistics due to potential population stratification in our datasets. Age, sex, and body mass index were all adjusted in regression models of GLGC, MAGIC and UK Biobank, and age and sex were also corrected in CKDGen (Dupuis et al., 2010; Soranzo et al., 2010; Willer et al., 2013; Ehret et al., 2016; Pattaro et al., 2016) (Supplementary Table S1).

### Study Design

**FIGURE 1:**
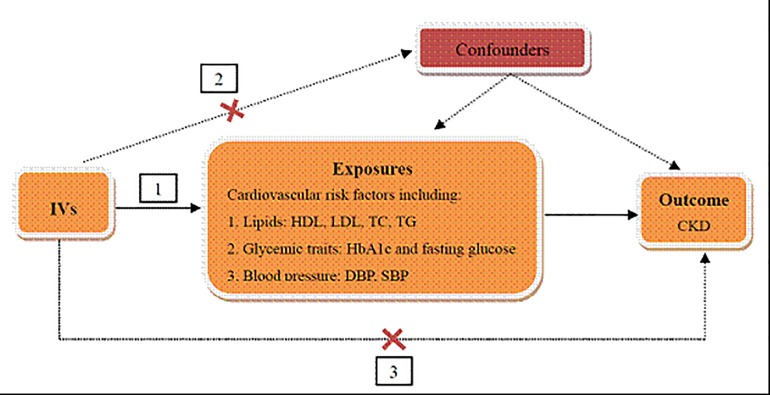
Schematic representation of TSMR analysis. Three assumptions of MR analysis are as follows: (1) IVs must be associated with cardiovascular risk factors, (2) IVs must not be associated with confounders, and (3) IVs must influence CKD only through cardiovascular risk factors. IVs, instrumental variables; HDL, high density lipoprotein; LDL, low density lipoprotein; TC, total cholesterol; TG, triglyceride; HbA1c, glycated hemoglobin; SBP, systolic blood pressure; DBP, diastolic blood pressure; CKD, chronic kidney disease.

The genetic variants used as IVs in TSMR analysis must satisfy three assumptions as follows (Figure 1): (1) IVs are strongly associated with cardiovascular risk factors, including lipids, glycemic traits, and blood pressure. (2) The IVs are independent of any known confounders. (3) The selected IVs are conditionally independent of CKD, given cardiovascular risk factors and confounders. The second and third assumptions are known as independence from pleiotropy (Bowden et al., 2015). In this study, we used cardiovascular risk factors as the exposures, including HDL cholesterol, LDL cholesterol, TC and TG for lipids, HbA1c and fasting glucose for glycemic traits, SBP and DBP for blood pressure, and CKD as the outcome to perform the TSMR analysis.

### IVs Selection and Validation

Instrumental variables must be associated with cardiovascular risk factors, including HDL cholesterol, LDL cholesterol, TC, TG, HbA1c, fasting glucose, SBP, and DBP. To ensure the close relationship between IVs and cardiovascular risk factors, we selected variants with *P* < 5 × 10^−8^ in the corresponding GWAS summary-level dataset. In addition, pairwise-linkage disequilibrium (LD) was calculated by PLINK 1.90 (Purcell et al., 2007) to ensure the independence among selected IVs and SNPs with *r*^2^> 0.001 will be removed from our analysis.

Selected IVs should be independent of any known confounders and conditionally independent of CKD, given the related traits of cardiovascular risk factors. The two assumptions indicate that IVs must influence CKD only through cardiovascular risk factors rather than another pathway (Geng et al., 2018). Firstly, we obtained the corresponding effect estimates of these variables on CKD. For the SNPs that were not available in the CKD, we used proxy SNPs that were highly correlated (*r*^2^ > 0.8) based on the SNP Annotation and Proxy (SNAP) search system^2^ (Johnson et al., 2008). Secondly, MR-Egger regression was performed to assess the horizontal pleiotropic (Bowden et al., 2015), which will be introduced in the following statistic analysis. Additionally, we excluded any palindromic SNPs that have minor allele frequency above 0.42 to ensure that the effects of the SNPs on the exposures correspond to the same allele as their effects on CKD (Davey Smith and Hemani, 2014). Moreover, to adjust for potential confounding, GWAS Catalog was used to check for the associations between selected IVs, smoking and type II diabetes (H1bAc and fasting glucose were excluded). Additionally, inspired by the idea of Palmer et al. (2012), we used the F statistic to investigate the association of selected IVs with the exposure on a web application^3^.

### Pleiotropy Assessment

Mendelian randomization-Egger regression was performed to assess the horizontal pleiotropic pathway between IVs and CKD, independent of cardiovascular risk factors (Bowden et al., 2015). MR-Egger regression was developed from Egger regression, which has been used to examine the publication bias in meta-analysis (ref needed here). This approach is expressed as α_i_ = βγ_i_ +β_0_, α_i_ represents the estimated effect between IVs and CKD; γ_i_ represents the estimated effect between IVs and cardiovascular risk factors, including HDL cholesterol, LDL cholesterol, TC, TG, HbA1c, fasting glucose, SBP, and DBP; slope β represents the estimated causal effect of cardiovascular risk factors on CKD; intercept β_0_ could be explained as the estimated average value of horizontal pleiotropic. Intercept with *P* > 0.05 indicates no horizontal pleiotropic exists. Additionally, the slope estimate provides the pleiotropy-corrected causal effect. However, this estimate may be underpowered if the selected SNPs collectively fail to explain a large proportion of the variance in the exposure (Bowden et al., 2015).

### TSMR Analysis

In this study, IVW method was used for TSMR analysis to estimate the causal effect between cardiovascular risk factors and CKD (Burgess et al., 2013). The causal effect β was estimated as *w*_i_(α_i_/γ_i_), where *i* refers to the *i*th IV, α_i_ defines as the association effect of IVs on CKD, γ_i_ represents the association effect of IVs on cardiovascular risk factors, and *w*_i_ means the weights of the causal effect of cardiovascular risk factors on CKD. MR Steiger test was also performed to infer the causal direction between exposures and CKD. It calculates the variance explained in the exposures and outcome by the instrumenting SNPs, and tests if the variance in the outcome is less than the exposures. Given the multiple testing situation, we used a conservative approach and applied a Bonferroni corrected significance level of 0.006 (0.05/8). 0.006 < *P* < 0.05 was considered as suggestive evidence for a potential association.

### Robust Adjusted Profile Score

The TSMR might fail if the selected SNPs are weak instruments. Therefore, we carried out a recently proposed method called robust adjusted profile score (RAPS) (Zhao et al., 2019) which considers the measurement error in SNP-exposure effects and is unbiased even when there are many (e.g., hundreds of) weak instruments, and is robust to systematic and idiosyncratic pleiotropy. Detailed information about this method please refer to the original paper (Zhao et al., 2019).

### Positive Control and Negative Control

Previous studies already reported established causal relationship between coronary artery disease (CAD) and CKD (Jansen et al., 2014), while there is little evidence indicating that cardiovascular risk factors are associated with myopia. To further demonstrate the validity of the selected IVs, we included coronary heart disease and myopia as positive and negative controls in our analysis. The summary statistics of them were respectively derived from CARDIoGRAMplusC4D consortium (Nikpay et al., 2015) and UK Biobank imputed genotype data^1^, including 184, 305 and 335, 700 individuals from European population.

### Sensitivity Analysis of TSMR

In the current study, weighted median and simple median methods were also applied as the follow-up sensitivity analysis (Geng et al., 2018). Compared with IVW, the weighted median and simple median methods have greater robustness to individual genetics with strongly outlying causal estimates and would generate a consistent estimate of the causal effect when valid IVs exceed 50% (Geng et al., 2018; Hemani et al., 2018). Furthermore, leave-one-out sensitivity analysis was performed to identify if the association was disproportionately influenced by a single SNP. The TSMR analysis is performed again but leaving out each SNP in turn and the overall analysis including all SNPs was shown for comparison (Mokry et al., 2016). All of the analysis was implemented by the “TwoSampleMR” package in R software environment.

## Results

### Selection and Validation of IVs

We obtained 84, 66, 78, 54, 10, 31, 103, 111 LD-independent (*r*^2^ < 0.001) IVs in total that achieved genome-wide significance (*P* < 5 × 10^−8^) from HDL cholesterol, LDL cholesterol, TC, TG, HbA1c, fasting glucose, SBP and DBP datasets, respectively. Not all of the SNPs were directly found in the CKD dataset; detailed information of all independent IVs in this TSMR analysis were shown in Supplementary Table S2. In addition, the intercept term, estimated for the exposures from MR-Egger regression, demonstrated that no horizontal pleiotropic exists in our TSMR analysis (Table 1). Moreover, we did not detect a direct association between selected IVs and smoking or type II diabetes (H1bAc and fasting glucose were excluded) in GWAS Catalog.

**Table 1:** Mendelian randomization (MR)-Egger regression intercepts.

Exposures	Outcome	MR-Egger regression	
		intercepts (95% CI)	*P*-value
HDL cholesterol	CKD	−0.005 (−0.014, 0.004)	0.282
LDL cholesterol	CKD	0.007 (−0.001, 0.016)	0.080
TC	CKD	0.002 (−0.007, 0.011)	0.687
TG	CKD	0.006 (−0.004, 0.016)	0.241
HbA1c	CKD	−0.006 (−0.034, 0.023)	0.707
Fasting glucose	CKD	−0.006 (−0.021, 0.01)	0.463
DBP	CKD	−0.003 (−0.018, 0.013)	0.746
SBP	CKD	−0.006 (−0.022, 0.011)	0.503

*A significant result (P > 0.05) indicates that the y-intercept of the MR-Egger regression line is not significantly different from zero and thus no pleiotropy exists. MR, mendelian randomization; HDL, high density lipoprotein; CKD, chronic kidney disease; LDL, low density lipoprotein; TC, total cholesterol; TG, triglyceride; SBP, systolic blood pressure; DBP, diastolic blood pressure; CI, confidence interval.*

F statistics were presented to demonstrate the strength of relationship between IVs and exposures, and F statistics greater than 10 are often considered as strong enough to mitigate against any bias of the causal IV estimate. Our selected IVs showed strong strength with F statistics ranging between 802 and 9447 (Supplementary Table S3).

### TSMR Analysis

**FIGURE 2:**
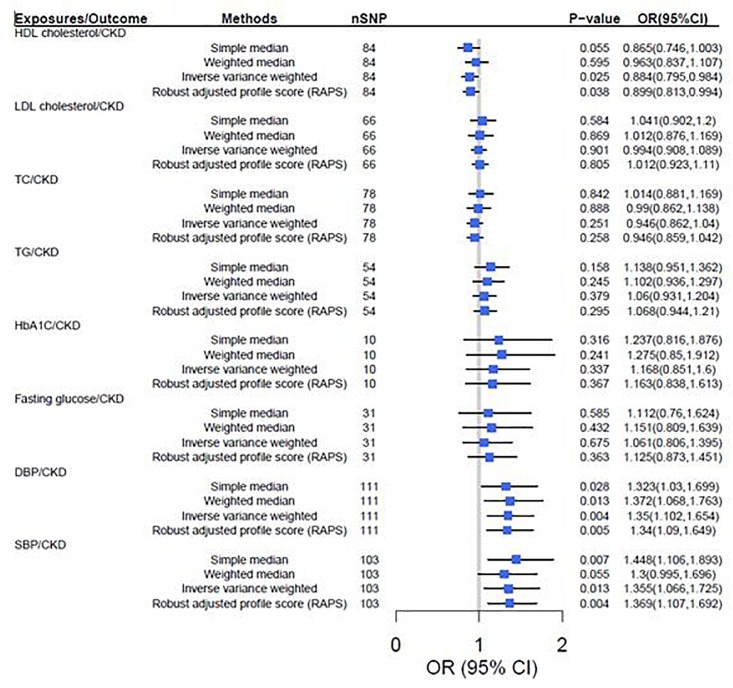
Two-sample mendelian randomization of cardiovascular risk factors and the risk of CKD. CKD was defined as eGFRcrea < 60 mL/min/1.73 m^2^. We used the 1 SD value from HDL cholesterol, LDL cholesterol, TC, TG, HbA1c, fasting glucose, SBP and DBP GWAS summary-level statistics. Results are standardized to a 1-SD increase in exposures; CI, confidence interval; HDL, high density lipoprotein; IVs, instrumental variables; CKD, chronic kidney disease; LDL, low density lipoprotein; TC, total cholesterol; TG, triglyceride; SBP, systolic blood pressure; DBP, diastolic blood pressure.

According to the IVW analysis results, the odds ratio (OR) and 95% confidence interval (CI) per 1-SD increase of DBP within CKD was 1.35 (1.10, 1.65) (*P* = 0.004). There was suggestive evidence for potential associations between genetically predicted higher HDL cholesterol [OR: 0.88, 95%CI (0.80, 0.98), *P* = 0.025] and lower adds of CKD, and between higher SBP [OR: 1.36, 95%CI (1.07, 1.73); *P* = 0.013] and higher adds of CKD. However, genetically predicted LDL cholesterol, TC, TG, HbA1c and fasting glucose were not associated with CKD (Figure 2).

### Robust Adjusted Profile Score

The results turned out to be consistent with the TSMR results that increased HDL cholesterol decreases the risk of CKD [OR = 0.89, 95% CI (0.79, 0.98), *P* = 0.025], but increased SBP [OR = 1.37, 95% CI (1.17, 1.56), *P* = 0.002] and DBP [OR = 1.33, 95% CI (1.14, 1.51), *P* = 0.003] increases the risk of CKD (Figure 2).

### MR Steiger Directionality Test

The inferred causal direction between exposures (HDL cholesterol, LDL cholesterol, TC, TG, HbA1c, fasting glucose, DBP, SBP) and CKD were “TRUE” in our MR Steiger test (Supplementary Table S4).

### Sensitivity Analysis of TSMR

In sensitivity analysis, there was suggestive evidence for potential associations between higher genetically predicted DBP [Weighted median: OR = 1.37, *P* = 0.013, 95% CI (1.07, 1.76); Simple median: OR = 1.32, *P* = 0.028, 95% CI (1.03, 1.70)] and SBP [Weighted median: OR = 1.30, *P* = 0.050, 95% CI (1.10, 1.70); Simple median: OR = 1.45, *P* = 0.007, 95% CI (1.11, 1.89)] and higher odds of CKD. However, genetically predicted HDL cholesterol, LDL cholesterol, TC, TG, HbA1c and fasting glucose were not associated with CKD (Figure 2). Furthermore, leave-one-out analysis showed a consistent significant causal effect of cardiovascular risk factors on CKD, supporting the robustness of our IVW analysis findings (Supplementary Table S5).

### Positive and Negative Control

Consistent with the conclusion of previous published paper (Jansen et al., 2014), our analysis demonstrated a causal relationship between all exposures and the positive control (coronary artery disease). As for negative control (myopia), our analysis showed no causal relationship between all exposures and myopia (Supplementary Table S6). Independent IVs of CAD and myopia were listed in Supplementary Tables S7, S8, respectively. Both negative and positive control analysis results suggest that the selected IVs of CKD are appropriate.

## Discussion

In the current study, by using genetic variants associated with cardiovascular risk factors as proxies, our TSMR analysis confirmed the causal association between DBP and increased risk of CKD. Genetically predicted SBP showed suggestive evidence for a possible causal association with CKD. We also detected suggestive evidence for an inverse causal association between genetically predicted HDL cholesterol and CKD. However, no evidence was found to support the associations between genetically predicted LDL cholesterol, TC, triglycerides, HbA1c, fasting glucose, and CKD.

Our conclusion of the causal relationship between LDL cholesterol, TC and CKD was consistent with the results of Lanktree et al. (2018). They also reported MR evidence for the association between genetically predicted higher HDL cholesterol and lower odds of CKD, but we did not draw the same conclusion in our sensitivity analysis (Lanktree et al., 2018). This inconsistency may be caused by a different selection of IVs and different analysis methods applied. Previous MR analysis, based on the GLGC and CKDGen dataset, reported a causal effect of HDL cholesterol on kidney function (Coassin et al., 2016). However, our study focused on CKD (eGFR < 60 mL/min/1.73 m^2^) rather than kidney function, which may be more directional compared with eGFR, due to the CKD’s clearly stages definition in clinical diagnosis(Coassin et al., 2016). Another MR analysis performed in a Chinese population demonstrated a causation between T2D and CKD, but we did not detect a significant causal relationship between glycemic traits (HbA1c and fasting glucose) and CKD in a European population (Xu et al., 2016), which demonstrates the importance of ethnicity in MR analysis.

Observational studies demonstrated that hypertension is almost invariably present in patients with renal failure, which is a powerful risk factor for cardiovascular diseases and CKD (Schiffrin et al., 2007). The renin–angiotensin system and sympathetic nervous system have been considered as important mechanisms involved in the elevation of blood pressure in subjects with CKD (Guyton and Coleman, 1999). The elevation of plasma catecholamine might increase nerve sympathetic traffic in CKD. Serving as a regulator of blood pressure (Converse et al., 1992; Neumann et al., 2004), renalase could metabolize catecholamines through dopamine, epinephrine, and norepinephrine (Xu et al., 2005). Investigators have discovered that renalase is an oxidase expressed mainly in glomeruli and proximal tubules of the kidney and cardiomyocytes (Vaziri et al., 2002). All the detected potential mechanisms suggest the relationship between blood pressure, cardiovascular disease and CKD, but the mechanism remains unclear (Schiffrin et al., 2007). Our current study successfully detected a causal relationship between genetically predicted blood pressure and CKD, which may provide novel evidence to further explain the mechanism of hypertension in CKD.

There are several strengths in our current study. First, we included multiple cardiovascular risk factors (8 in total) as the exposures, hence we were able to include a relatively large number of IVs. Then, to provide relatively consistent causal effect estimates, we also performed sensitivity analysis using several different approaches. In addition, we used a web application^3^ to investigate the magnitude of bias arising from sample overlap with a conservative value of concentration parameter, and it would not be substantial due to sample overlap in our study. Furthermore, CAD and myopia were used as positive and negative controls, respectively, to demonstrate the validity of selected IVs. However, this study also has some limitations. Firstly, as we only used summary statistics and had no access to the original individual clinical outcome measures, we could not conduct analyses stratified by subtypes of CKD. Secondly, different standards of quality control in individual-level GWAS may affect our results. Therefore, the results cannot be easily generalized.

Using a genetic approach, we found DBP is causally associated with CKD risk. Furthermore, we provided suggestive evidence that SBP is causally associated with CKD risk and HDL cholesterol is inversely causally associated with CKD. However, additional human and animal studies are still needed to further confirm our TSMR results.

## Author Contributions

H-ML and H-WD: research idea and study design. H-ML: data acquisition, statistical analysis, and writing the manuscript. H-ML, H-WD, and QH: data analysis and interpretation. G-YS and H-MX: supervision or mentorship. H-ML, QZ, B-YL, W-DS, XQ, and W-QL: new manuscript revision and point to point response to the reviewers. All authors read and approved the final manuscript.

## Conflict of Interest Statement

The authors declare that the research was conducted in the absence of any commercial or financial relationships that could be construed as a potential conflict of interest.

## Footnotes

1 http://www.nealelab.is/blog/2017/9/11/details-and-considerations-of-the-uk-biobank-gwas

2 http://www.broadinstitute.org/mpg/snap/ldsearchpw.php

3 https://sb452.shinyapps.io/overlap

## Abbreviations

CI: confidence interval
CKD: chronic kidney disease
DBP: diastolic blood pressure
eGFR: estimated glomerular filtration rate
GWAS: genome wide association study
HbA1c: glycated hemoglobin
HDL: high density lipoprotein
IVs: instrumental variables
IVW: Inverse variance weighted
LD: linkage disequilibrium
LDL: low density lipoprotein
MR: mendelian randomization
OR: odds ratio
SBP: systolic blood pressure
TC: total cholesterol
TG: triglyceride
TSMR: two-sample mendelian randomization.

## References

[B1] Boef A. G. Dekkers O. M. Le Cessie S. (2015). Mendelian randomization studies: a review of the approaches used and the quality of reporting. *Int. J. Epidemiol.* 44 496–511. 10.1093/ije/dyv071 25953784

[B2] Bowden J. Davey Smith G. Burgess S. (2015). Mendelian randomization with invalid instruments: effect estimation and bias detection through egger regression. *Int. J. Epidemiol.* 44 512–525. 10.1093/ije/dyv080 26050253 PMC4469799

[B3] Burgess S. Butterworth A. Thompson S. G. (2013). Mendelian randomization analysis with multiple genetic variants using summarized data. *Genet. Epidemiol.* 37 658–665. 10.1002/gepi.21758 24114802 PMC4377079

[B4] Ceriello A. De Cosmo S. Rossi M. C. Lucisano G. Genovese S. Pontremoli R. (2017). Variability in HbA1c, blood pressure, lipid parameters and serum uric acid, and risk of development of chronic kidney disease in type 2 diabetes. *Diabetes Obes. Metab.* 19 1570–1578. 10.1111/dom.12976 28432733

[B5] Coassin S. Friedel S. Kottgen A. Lamina C. Kronenberg F. (2016). Is high-density lipoprotein cholesterol causally related to kidney function? evidence from genetic epidemiological studies. *Arterioscler. Thromb. Vasc. Biol.* 36 2252–2258. 10.1161/ATVBAHA.116.308393 27687604 PMC5084637

[B6] Converse R. L. Jr. Jacobsen T. N. Toto R. D. Jost C. M. Cosentino F. Fouad-Tarazi F. (1992). Sympathetic overactivity in patients with chronic renal failure. *N. Engl. J. Med.* 327 1912–1918. 10.1056/NEJM199212313272704 1454086

[B7] Davey Smith G. Hemani G. (2014). Mendelian randomization: genetic anchors for causal inference in epidemiological studies. *Hum. Mol. Genet.* 23 R89–R98. 10.1093/hmg/ddu328 25064373 PMC4170722

[B8] Dupuis J. Langenberg C. Prokopenko I. Saxena R. Soranzo N. Jackson A. U. (2010). New genetic loci implicated in fasting glucose homeostasis and their impact on type 2 diabetes risk. *Nat. Genet.* 42 105–116. 10.1038/ng.520 20081858 PMC3018764

[B9] Ehret G. B. Ferreira T. Chasman D. I. Jackson A. U. Schmidt E. M. Johnson T. (2016). The genetics of blood pressure regulation and its target organs from association studies in 342,415 individuals. *Nat. Genet.* 48 1171–1184. 10.1038/ng.3667 27618452 PMC5042863

[B10] Emdin C. A. Khera A. V. Kathiresan S. (2017). Mendelian randomization. *Jama* 318 1925–1926. 10.1001/jama.17219 29164242

[B11] Geng T. Smith C. E. Li C. Huang T. (2018). Childhood BMI and adult type 2 diabetes, coronary artery diseases, chronic kidney disease, and cardiometabolic traits: a mendelian randomization analysis. *Diabetes care* 41 1089–1096. 10.2337/dc17-2141 29483184

[B12] Guyton A. C. Coleman T. G. (1999). Quantitative analysis of the pathophysiology of hypertension 1969. *J. Am. Soc. Nephrol.* 10 2248–2258. 10505704

[B13] Hager M. R. Narla A. D. Tannock L. R. (2017). Dyslipidemia in patients with chronic kidney disease. *Rev. Endocr. Metab. Disord.* 18 29–40. 10.1007/s11154-016-9402-z 28000009

[B14] Hall M. E. do Carmo J. M. da Silva A. A. Juncos L. A. Wang Z. Hall J. E. (2014). Obesity, hypertension, and chronic kidney disease. *Int. J. Nephrol. Renovas. Dis.* 7 75–88. 10.2147/IJNRD.S39739 PMC3933708 24600241

[B15] Hemani G. Zheng J. Elsworth B. Wade K. H. Haberland V. Baird D. (2018). The MR-Base platform supports systematic causal inference across the human phenome. *elife* 7:e34408. 10.7554/eLife.34408 29846171 PMC5976434

[B16] Heywood J. T. Fonarow G. C. Costanzo M. R. Mathur V. S. Wigneswaran J. R. Wynne J. (2007). High prevalence of renal dysfunction and its impact on outcome in 118,465 patients hospitalized with acute decompensated heart failure: a report from the ADHERE database. *J. Card. Fail.* 13 422–430. 10.1016/j.cardfail.2007.03.011 17675055

[B17] Hill N. R. Fatoba S. T. Oke J. L. Hirst J. A. O’Callaghan C. A. Lasserson D. S. (2016). Global prevalence of chronic kidney disease - a systematic review and meta-analysis. *PLoS One* 11:e0158765. 10.1371/journal.pone.0158765 27383068 PMC4934905

[B18] Jansen H. Samani N. J. Schunkert H. (2014). Mendelian randomization studies in coronary artery disease. *Eur. Heart J.* 35 1917–1924. 10.1093/eurheartj/ehu208 24917639

[B19] Johnson A. D. Handsaker R. E. Pulit S. L. Nizzari M. M. O’Donnell C. J. de Bakker P. I. (2008). SNAP: a web-based tool for identification and annotation of proxy SNPs using HapMap. *Bioinformatics* 24 2938–2939. 10.1093/bioinformatics/btn564 18974171 PMC2720775

[B20] Katan M. B. (2004). Apolipoprotein E isoforms, serum cholesterol, and cancer. 1986. *Int. J. Epidemiol.* 33:9. 10.1093/ije/dyh312 15075136

[B21] Lanktree M. B. Theriault S. Walsh M. Walsh M. Paré G. (2018). HDL cholesterol, LDL cholesterol, and triglycerides as risk factors for CKD: a mendelian randomization study. *Am. J. Kidney Dis.* 71 166–172. 10.1053/j.ajkd.2017.06.011 28754456

[B22] Lawlor D. A. Harbord R. M. Sterne J. A. Timpson N. Davey Smith G. (2008). Mendelian randomization: using genes as instruments for making causal inferences in epidemiology. *Stat. Med.* 27 1133–1163. 10.1002/sim.3034 17886233

[B23] Mokry L. E. Ross S. Timpson N. J. Sawcer S. Davey Smith G. Richards J. B. (2016). Obesity and multiple sclerosis: a mendelian randomization study. *PLoS Med.* 13:e1002053. 10.1371/journal.pmed.1002053 27351487 PMC4924848

[B24] Neumann J. Ligtenberg G. Klein I. I. Koomans H. A. Blankestijn P. J. (2004). Sympathetic hyperactivity in chronic kidney disease: pathogenesis, clinical relevance, and treatment. *Kidney Int.* 65 1568–1576. 10.1111/j.1523-1755.2004.00552.x 15086894

[B25] Nikpay M. Goel A. Won H. H. Hall L. M. Willenborg C. Kanoni S. (2015). A comprehensive 1,000 genomes-based genome-wide association meta-analysis of coronary artery disease. *Nat. Genet.* 47 1121–1130. 10.1038/ng.3396 26343387 PMC4589895

[B26] Palmer T. M. Lawlor D. A. Harbord R. M. Sheehan N. A. Tobias J. H. Timpson N. J. (2012). Using multiple genetic variants as instrumental variables for modifiable risk factors. *Stat. Methods Med. Res.* 21 223–242. 10.1177/0962280210394459 21216802 PMC3917707

[B27] Pattaro C. Teumer A. Gorski M. Chu A. Y. Li M. Mijatovic V. (2016). Genetic associations at 53 loci highlight cell types and biological pathways relevant for kidney function. *Nat. Commun.* 7:10023. 10.1038/ncomms10023 26831199 PMC4735748

[B28] Purcell S. Neale B. Todd-Brown K. Thomas L. Ferreira M. A. Bender D. (2007). PLINK: a tool set for whole-genome association and population-based linkage analyses. *Am. J. Hum. Genet.* 81 559–575. 10.1086/519795 17701901 PMC1950838

[B29] Romagnani P. Remuzzi G. Glassock R. Levin A. Jager K. J. Tonelli M. (2017). Chronic kidney disease. *Nat. Rev. Dis. Primers* 3:17088. 10.1038/nrdp.2017.88 29168475

[B30] Schiffrin E. L. Lipman M. L. Mann J. F. (2007). Chronic kidney disease: effects on the cardiovascular system. *Circulation* 116 85–97. 17606856 10.1161/CIRCULATIONAHA.106.678342

[B31] Soranzo N. Sanna S. Wheeler E. Gieger C. Radke D. Dupuis J. (2010). Common variants at 10 genomic loci influence hemoglobin A(1)(C) levels via glycemic and nonglycemic pathways. *Diabetes* 59 3229–3239. 10.2337/db10-0502 20858683 PMC2992787

[B32] Vaziri N. D. Ni Z. Oveisi F. Liang K. Pandian R. (2002). Enhanced nitric oxide inactivation and protein nitration by reactive oxygen species in renal insufficiency. *Hypertension* 39 135–141. 10.1161/hy0102.100540 11799092

[B33] Willer C. J. Schmidt E. M. Sengupta S. Peloso G. M. Gustafsson S. Kanoni S. (2013). Discovery and refinement of loci associated with lipid levels. *Nat. Genet.* 45 1274–1283. 10.1038/ng.2797 24097068 PMC3838666

[B34] Xu J. Li G. Wang P. Yao X. Li Y. Wu Y. (2005). Renalase is a novel, soluble monoamine oxidase that regulates cardiac function and blood pressure. *J. Clin. Invest.* 115 1275–1280. 10.1172/JCI24066 15841207 PMC1074681

[B35] Xu M. Bi Y. Huang Y. Xie L. Hao M. Zhao Z. (2016). Type 2 diabetes, diabetes genetic score and risk of decreased renal function and albuminuria: a mendelian randomization study. *Ebiomedicine* 6 162–170. 10.1016/j.ebiom.2016.02.032 27211558 PMC4856750

[B36] Zhao J. Hemani G. Bowden J. Small D. S. (2019). Statistical inference in two-sample summary-data mendelian randomization using robust adjusted profile score. arXiv:1801.09652 [Preprint].

